# CRISPR-Based Editing of the *Medicago truncatula LEC1* Gene

**DOI:** 10.3390/plants13223226

**Published:** 2024-11-16

**Authors:** Elina A. Potsenkovskaia, Varvara E. Tvorogova, Veronika Y. Simonova, Zakhar S. Konstantinov, Anna S. Kiseleva, Andrew G. Matveenko, Anna V. Brynchikova, Ludmila A. Lutova

**Affiliations:** 1 Department of Genetics and Biotechnology, Saint Petersburg State University, 7/9 Universitetskaya Embankment, 199034 Saint Petersburg, Russia; v.tvorogova@spbu.ru (V.E.T.); zakhar.konstantinov25@gmail.com (Z.S.K.); a.matveenko@spbu.ru (A.G.M.); la.lutova@gmail.com (L.A.L.); 2 Plant Biology and Biotechnology Department, Sirius University of Science and Technology, 1 Olympic Avenue, 354340 Sochi, Russia; nikasimonova14@gmail.com (V.Y.S.); anykisely@gmail.com (A.S.K.); annbv19@gmail.com (A.V.B.); 3 Center for Genetic Technologies, N. I. Vavilov All-Russian Institute of Plant Genetic Resources (VIR), 42 Bolshaya Morskaya Street, 190000 Saint Petersburg, Russia

**Keywords:** CRISPR, LEC1, MtNF-YB10, *Medicago truncatula*, agrobacterial transformation, regeneration, somatic embryogenesis

## Abstract

*Arabidopsis thaliana LEAFY COTYLEDON1 (LEC1)* gene is shown to have numerous diverse functions in plant development, including the regulation of embryo morphogenesis and maturation, hypocotyl elongation, flowering transition, etc. However, the functions of *LEC1* orthologs in different plant species have not been extensively studied. In this study, we obtained a line of *Medicago truncatula*, a model leguminous plant, carrying the loss-of-function mutation in the *MtLEC1 (MtNF-YB10)* gene, orthologous to *LEC1*, using the Clustered Regularly Interspaced Short Palindromic Repeats/CRISPR-associated proteins (CRISPR/Cas9) genome editing system. Edited plants with loss of *MtNF-YB10* function did not demonstrate any severe abnormalities during their normal growth and gave viable seeds, but their capability for somatic embryogenesis in vitro was dramatically reduced. The T1 progeny of unedited plants with a Cas9-gRNA cassette insertion was also analyzed based on the suggestion that editing could occur during seed formation. However, no edited plants were found in the T1 generation. These results suggest divergent functions of *LEC1* orthologs and make it possible to investigate potential specific *MtNF-YB10* functions.

## 1. Introduction

The first plant genetic studies were carried out based on the detection of existing mutations or the induction of chemical or radiation mutagenesis. These types of mutagens often induce random mutations across the genome, which can lead to unintended consequences, including harmful mutations that may compromise organism viability or fitness. Radiation causes clustered DNA damage that is often difficult for cells to repair accurately. This can lead to a higher frequency of errors during DNA replication, resulting in mutation patterns that are complex and difficult to manage [1]. In addition to direct DNA damage, chemical mutagens can induce epigenetic changes that may not be immediately observable but can influence gene expression over generations [2]. Both chemical and radiation mutagenesis face stringent regulatory scrutiny due to potential health risks associated with exposure to mutagens. Compliance with these regulations can slow down research progress and increase costs [3]. With the advent of modern genome editing technologies, precise genetic modifications in the cells of many species have become possible. Genome editing provides the ability to precisely modify DNA by adding, removing, or changing genetic material. To edit the plant genome, methods based on Clustered Regularly Interspaced Short Palindromic Repeats/CRISPR-associated proteins (CRISPR/Cas) [4], Transcription Activator-Like Effector Nucleases, Zinc-Finger Nucleases [5], and Oligo-Directed Mutagenesis [6] are used. The development of these technologies has expanded the possibilities of genome modification for studying gene function, metabolic engineering, and crop improvement [7].

Currently, the most widely used editing system is CRISPR/Cas technology, which has been successfully applied to more than one hundred plant species. CRISPR/Cas technology is based on the RNA-targeted cleavage of one or both DNA strands by Cas endonuclease, followed by DNA repair by intrinsic cell mechanisms. CRISPR/Cas provides the ability to make targeted modifications in specific DNA sequences in the plant genome to introduce desired traits. In addition to offering high flexibility for targeting genomic regions and being relatively low-cost, this technology allows for the obtaining of progeny in subsequent generations that carry only the desired mutation without the *Cas* gene or other vector components integrated into the genome [8,9].

The *Agrobacterium tumefaciens*-mediated CRISPR/Cas9 genome editing system is now actively used not only on model objects but also on agricultural species to study and improve their agronomic properties. Its application has been reported for many important food crops, such as rice [10], maize [11], wheat [12], soybeans [13], and peas [14]. Genome editing provides opportunities to obtain varieties with valuable agronomic traits and to study molecular mechanisms of crop resistance to biotic and abiotic factors [15]. For example, the drought tolerance of plants has been investigated by knocking out target genes in different crops, including tomato [16], rice [17], and wheat [18]. Resistance of *Vitis vinifera* to fungal pathogens was also investigated with the application of editing technology [19]. 

For many plant species, introducing editing agents through agrobacterial transformation and further regeneration is the only possible option for obtaining edited plants. Therefore, species with low transformation efficiency are usually also difficult for genome editing. For example, most leguminous species are recalcitrant to transformation and, therefore, to genome editing. Unlike most legumes, *Medicago truncatula*, a popular model object for legume research, is transformed and edited rather easily. There are multiple reports on successful genome editing for this species. For example, mutant *M. truncatula* lines with changes in the profile of secondary metabolites have been generated by knocking out the *CYP93E2* gene, which is responsible for the biosynthesis of the triterpenoid soyasapogenol B [20]. Editing of multiple sites of a single gene has also been shown for *M. truncatula Hua enhancer1*, which encodes a protein involved in small RNA processing [21]. Approaches to optimize *M. truncatula* genome editing using different promoters and RNA processing strategies have been described. For instance, the editing frequency increased when the *Cas9* gene was driven by the *Arabidopsis UBQ10* promoter. As a result, the mutation efficiency reached 70% in the T0 generation [22]. The usage of a polycistronic gRNA expression system led to inheritable mutations in *M. truncatula*, simultaneously targeting *NCR53*, *NCR4*, and *NCR55*, which encode nodule-specific cysteine-rich peptides. In this study, gRNAs were expressed through the Pol II promoter (*CmYLCV* promoter) and processed into individual gRNAs by Csy4 endoribonuclease. This approach increased efficiency and speed of multiplex CRISPR/Cas9 editing [23]. Furthermore, multiplex CRISPR/Cas9-mediated mutagenesis allowed the induction of maternal haploid production in vivo in *M. truncatula* by the loss of function of *MtDMP8* and *MtDMP9*, homologues of *Zea mays DOMAIN OF UNKNOWN FUNCTION 679*, involved in reproductive development [24]. Using targeted mutagenesis with the expression of gRNA modules under the control of several *MtU6* promoters, Zhu et al. [25] generated triple knockout mutants by targeting *MtCEP1*, *MtCEP2*, and *MtCEP12*, which belong to the C-terminally Encoded Peptide gene family, thereby confirming their role in regulating lateral root development and symbiotic nodulation.

*M. truncatula* is one of a few leguminous plants with high potential for somatic embryogenesis (SE), which makes it possible for this species to obtain transgenic and edited plants. Therefore, studying genes regulating SE in this object can be useful for the search of SE regulators in legumes as a whole. 

One of the well-known SE regulators is the *LEAFY COTYLEDON1* (*LEC1*) gene. LEC1 and its homologue LEC1-like (*L1L*) both belong to a specific branch of the Nuclear Factor YB (NF-YB) transcription factor family, known as the LEC1-type group. LEC1-type group members are commonly involved in the regulation of seed development and embryogenesis [26]. A large-scale search for LEC1 targets in seeds has shown that it directly regulates the expression of various groups of genes at different stages of embryonic development, including genes related to embryonic morphogenesis, photosynthesis, and seed maturation [27]. 

*LEC1* roles and regulatory mechanisms have been studied primarily in *Arabidopsis thaliana*, but its orthologs have been identified in other plants, such as soybean [28], cassava [29], maize [30], rice [31], and *Brassica napus* [32], highlighting its evolutionary conservation and functional importance. *GmLEC1* regulates embryonic development and lipid biosynthesis in soybean, functioning similarly to its orthologs in *Arabidopsis* [28]. Overexpression of *MeLEC1* and *MeLEC2* can induce embryogenic characteristics, demonstrating their potential utility in improving transformation efficiency in recalcitrant genotypes [29]. The changes in fatty acid composition resulting from *BnLEC1* expression in transgenic canola seeds exemplify how genetic modification can enhance the nutritional quality and marketability of agricultural products [32].

Ectopic expression of *LEC1* is sufficient to induce SE in *Arabidopsis thaliana* [33]. *LEC1* loss-of-function mutant exhibits a pleiotropic phenotype including abnormal germination, seed desiccation intolerance, reserve accumulation defects, leafy cotyledons, and reduced hypocotyl elongation [33,34,35,36]. These studies, along with research on rice CRISPR mutants with *OsLEC1* loss of function [37], suggest that obtaining such mutants can be challenging due to the essential role of this gene in the embryo and seed development process. In our previous study, we have shown that the *LEC1* ortholog in *M. truncatula*, *MtNF-YB10*, is also expressed in seeds and during SE [38]. In the current research, we aimed to obtain plants with mutations in the *MtNF-YB10* gene using the CRISPR/Cas system.

## 2. Results

### 2.1. Obtaining Plants with Edited MtNF-YB10 Gene

**Figure 1:**
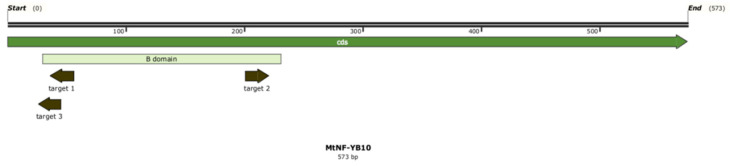
Map of the *MtNF-YB10* gene. The gene does not contain introns. A part of the gene encoding B domain, conservative for NF-YB proteins, is shown in pale green. Targets 1, 2, and 3 are marked with dark green arrows, pointing to PAM. Figure 1 was generated in Snapgene v6.2.1.

To obtain a vector for *MtNF-YB10* editing, we used the vector construction system developed by Xing et al. [39]. We designed two different targets for *MtNF-YB10* editing: GATACGAATCACGTTTGCTAT (target 1) and GAAGATATACTATGGGCAAT (target 2) (Figure 1). The pHSE401 vector served as a backbone, and the resulting plasmid contained two gRNA genes with these two targets under the control of U6-26 and U6-29 promoters, respectively. For both genes, U6-26 terminators were used.

Next, we performed agrobacterial transformation of the *M. truncatula* R108 line with this plasmid. As a result, 22 regenerated plants were obtained. Sanger sequencing of PCR products from the *MtNF-YB10* loci in these plants indicated that apparently none of them contained edited alleles, resulting in an editing efficiency of 0% for this system and these targets. In a parallel experiment, we successfully obtained R108 plants with edited alleles of a different gene using the same editing and transformation technique, suggesting that the lack of edited plants in the case of *MtNF-YB10* may be due to target- or gene-specific characteristics.

According to CRISPR-P v.2.0 [40], the on-target efficiency scores of targets 1 and 2 were 0.3632 and 0.0343, respectively. We decided to perform the second experiment with a single target GTTTGCTATTGGCATGTAC (target 3) (Figure 1), which has an on-target efficiency of 0.2615. For editing vector obtaining, the same cloning system was used [39], and the resulting plasmid contained one *gRNA* gene under the control of the U6-26 promoter and terminator.

**Figure 2:**
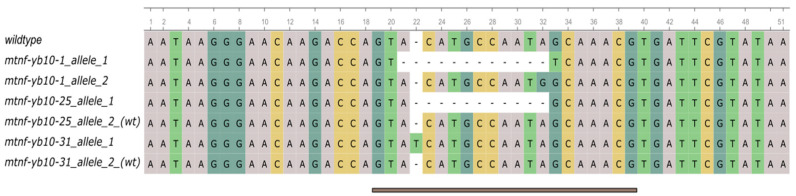
Alignment of sequences of edited and wildtype (wt) alleles identified from cloned PCR fragments from three T0 transgenic lines. Only aligned regions of interest are displayed; target without PAM is underlined. Figure 2 was generated in Ugene v48.1.

The transformation with the editing plasmid containing target 3 produced 41 regenerants. Sanger sequencing of PCR products obtained from the *MtNF-YB10* locus of these plants was performed. Most of the regenerants did not demonstrate any signs of editing. However, chromatograms from three plants displayed overlapping peaks starting at the target site, suggesting the presence of at least one potentially edited allele. The PCR products from three potentially edited plants were cloned in a pAL2-T vector, and individual clones were sequenced (Figure 2). The allele sequences obtained are presented in Table 1.

Thus, we obtained three transgenic plants with a *Cas9* insertion, each containing one edited allele causing a frameshift. With 3 plants out of 41 regenerants containing an edited allele, the editing efficiency for this target was 7.3%. The *mtnf-yb10-1* mutant, with a loss-of-function allele with a loss of 11 nucleotides and missense allele I13M, did not survive to flowering. The *mtnf-yb10-25* and *mtnf-yb10-31* plants produced seeds, and we proceeded to genotype their T1 offspring.

### 2.2. Analysis of the T1 Progeny of Unedited Plants

Apart from the 3 plants with edited *MtNF-YB10* alleles, we obtained 13 unedited transgenic T0 plants, which had transgenic insertion with *Cas9* and *gRNA* genes and, at the same time, produced seeds. We decided to check if the editing activity could manifest in the T0 progeny, for instance, during seed development, and lead to the *MtNF-YB10* editing in the T1 generation. 

We sowed 22 seeds from these 13 T0 unedited plants and then genotyped the germinated seedlings. As a result, we did not detect an edited *MtNF-YB10* allele in any of the seedlings genotyped.

### 2.3. Analysis of T1 and T2 Progeny from Edited Plants

#### 2.3.1. Allele Sequence Analysis of T1 Edited Plants

**Figure 3:**
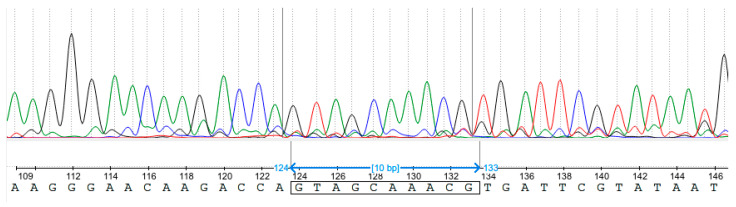
Sequencing chromatogram of a PCR fragment obtained from the *MtNF-YB10* locus of a T1 *mtnf-yb10-25-2* plant. The blue two-headed arrow marks part of the sequence corresponding to the target site. Different line colours correspond to different nucleotide types written in the bottom. Figure 3 was generated in Ugene v48.1.

We obtained two T1 plants from the *mtnf-yb10-31* regenerant and two T1 plants from the *mtnf-yb10-25* regenerant. Remarkably, the two T1 *mtnf-yb10-31* progeny plants died before flowering at the developmental stage with one simple leaf only. Only one of them was sequenced postmortem, while DNA from the second one was not isolated. Therefore, in total, three T1 plants were genotyped using Sanger sequencing of PCR fragments obtained from the *MtNF-YB10* locus and allele separation by Synthego ICE. Sequencing showed that among the progeny of the T0 plant *mtnf-yb10-25*, one T1 plant was heterozygous with one edited and one wildtype (wt) allele (*mtnf-yb10-25-1*), and another T1 plant was homozygous with two edited alleles (*mtnf-yb10-25-2*) (Figure 3). The single genotyped plant from the offspring of *mtnf-yb10-31* was also heterozygous, with one edited and one wt allele (*mtnf-yb10-31-1*) (Table 2).

#### 2.3.2. Analysis of Homozygous MtNF-YB10 Loss-of-Function Mutants

**Figure 4:**
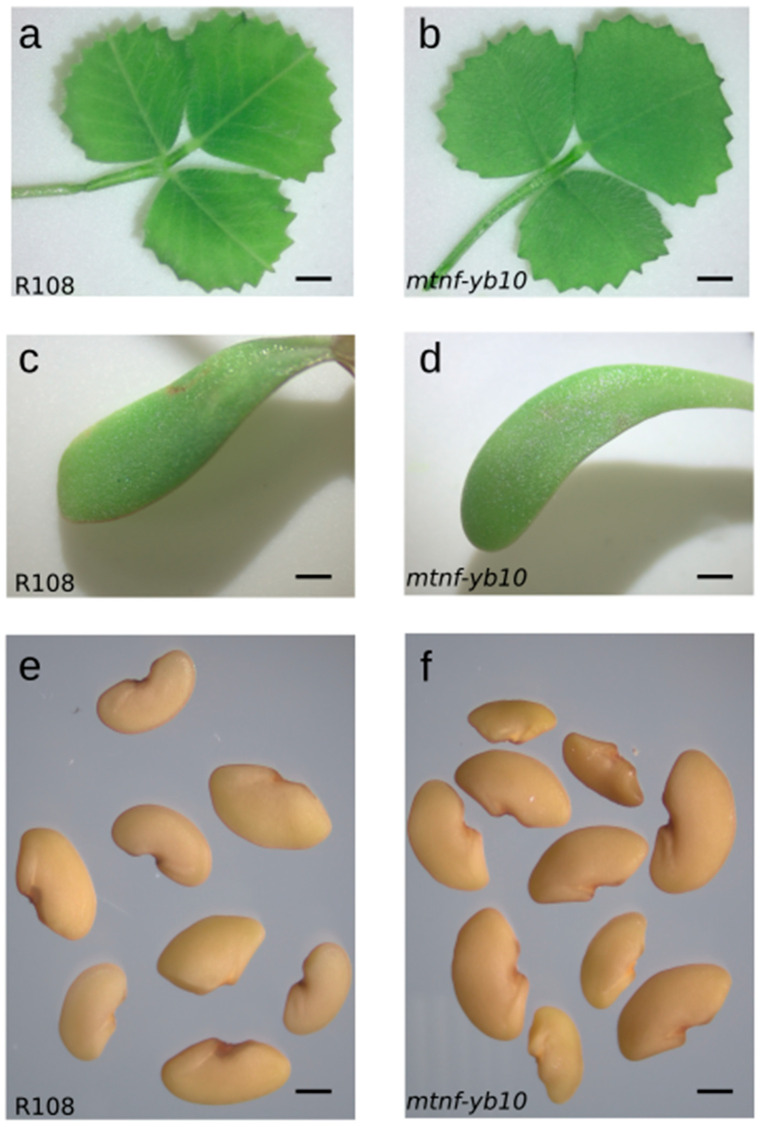
Phenotypic comparison of 34-day-old wt and mutant *mtnf-yb10-25-2* progeny plants and seeds. (**a**,**b**) True leaves of wt (**a**) and mutant (**b**) plants; (**c**,**d**) cotyledons of wt (**c**) and mutant (**d**) plants; (**e**,**f**) general view of wt (**e**) and mutant (**f**) seeds. Scale bars are 1 mm for (**a**,**b**), 2 mm for (**c**,**d**), and 1 mm for (**e**,**f**).

Interestingly, the *mtnf-yb10-25-2* plant, which was a homozygous *MtNF-YB10* loss-of-function mutant, did not demonstrate any visible phenotypic abnormalities in comparison with the R108 wt. It produced viable progeny with leaves that showed no visible abnormalities; the seedlings did not have leafy cotyledons (Figure 4a–d).

**Figure 5:**
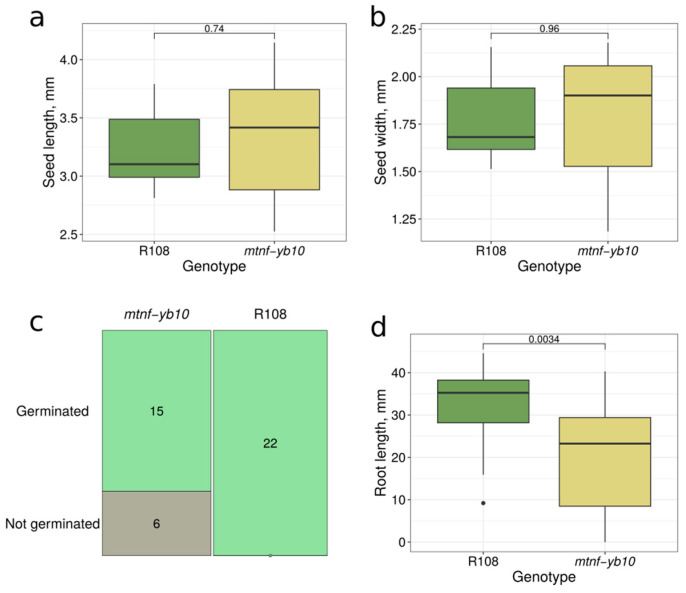
Evaluation of seed size, germination rate, and root length of wt and mutant *mtnf-yb10-25-2* progeny plants. (**a**,**b**) Boxplots representing seed length (**a**) and width (**b**) for wildtype R108 and mutant *mtnf-yb10* plants. Data were obtained for 7–10 seeds for different genotypes. To assess the statistical significance of the observed differences, the Wilcoxon signed-rank test was used. (**c**) Mosaic plot representing the number of germinated and not germinated seeds for different genotypes. The germination rate differed significantly between genotypes (*p*-value = 0.008901, Fisher test). (**d**) Boxplot representing root length for wt R108 and mutant *mtnf-yb10* seedlings. Data were obtained for 16–22 seedlings for different genotypes. To assess the statistical significance of the observed differences, the Wilcoxon signed-rank test was used.

We evaluated several phenotypic characteristics of the *mtnf-yb10-25-2* progeny. We did not detect statistically significant differences in seed size (Figure 5a,b). Furthermore, although the variance in seed size appeared to be higher in the *mtnf-yb10* genotype (Figure 4e,f), we also did not detect statistically significant differences between the wildtype and *mtnf-yb10* genotypes for this parameter (*p*-values were =0.2697 and 0.3714 for differences in variance in seed length and width, respectively, according to the Brown–Forsythe Levene-type test). 

Nevertheless, the germination rate of the *mtnf-yb10* seeds was significantly lower compared to the wildtype (Figure 5c), with *p*-value = 0.008901, Fisher test. The root length of the germinated seedlings was also reduced (Figure 5d).

**Figure 6:**
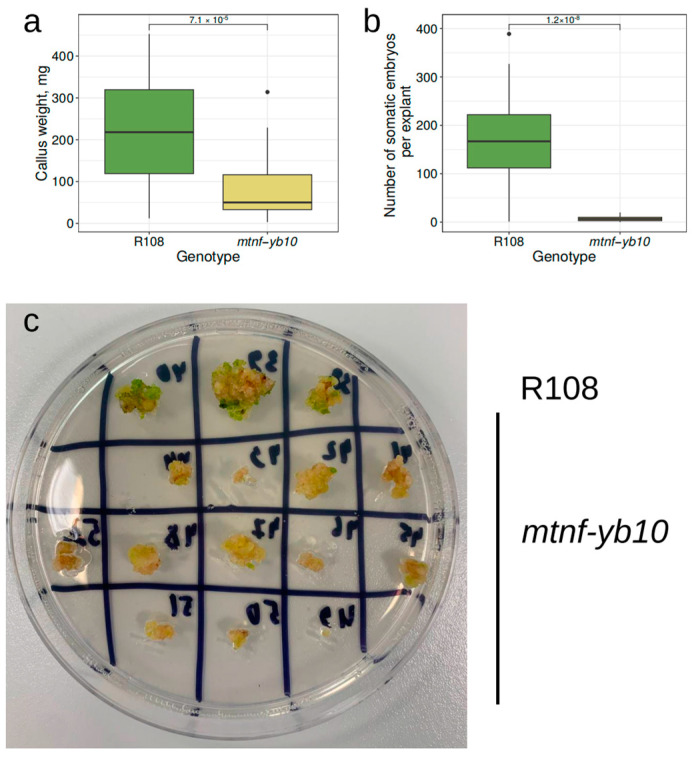
Evaluation of capacity for callus formation and SE for mutant *mtnf-yb10-25-2* progeny plants. (**a**,**b**) Boxplot representing callus weight (**a**) and number of somatic embryos (**b**) after in vitro cultivation of explants from wildtype R108 and mutant *mtnf-yb10* plants. Data were obtained from 24–27 explants for different genotypes; each explant was taken from the individual plant. To assess the statistical significance of the observed differences, the Wilcoxon signed-rank test was used. (**c**) Calli developed from explants taken from wildtype R108 and mutant *mtnf-yb10* plants on the 68th day of cultivation.

We also assessed the SE and callus formation capacity for *mtnf-yb10* plants, comparing them with control R108 plants. According to our results, the number of somatic embryos per explant as well as the weight of in vitro-formed calli were dramatically reduced (Figure 6). 

**Figure 7:**
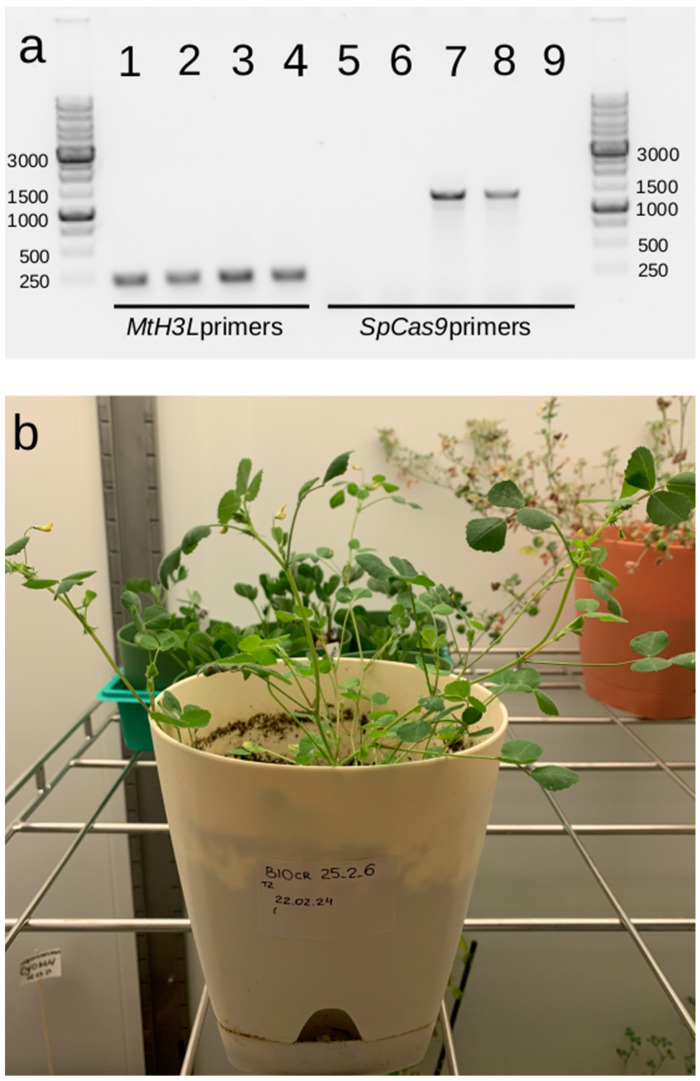
Analysis of T2 progeny of the *mtnf-yb10-25-2* plant. (**a**) PCR identification of *mtnf-yb10-25-2* progeny plants without insertion of the cassette with *Cas9* expression. 1,5—wt R108; 2,6—T2 plant without insertion of the cassette with Cas9 expression (*mtnf-yb10-25-2-6*); 3,4,7,8—two plants with insertion of the cassette with *Cas9* expression; 1,2,3,4—reference gene *MtH3L* fragments; 5,6,7,8—*SpCas9* fragments; 9—H_2_O (K-); margins—1 Kb SibEnzyme ladder; (**b**) T2 plant *mtnf-yb10-25-2-6* without insertion of the *Cas9* gene at the flowering stage.

Among the progeny of *mtnf-yb10-25-2*, an edited *mtnf-yb10-25-2-6* plant without insertion of the *Cas9* gene was identified, which also produced viable seeds (Figure 7).

## 3. Discussion

The obtaining of plant lines with edited genes that perform important functions can be challenging. In this study, we obtained a total of 63 plants that regenerated after transformation with the CRISPR plasmid for editing of the *MtNF-YB10* gene. Among them, only three plants had at least one edited allele of *MtNF-YB10*, which accounts for 4.7%. Interestingly, the first editing experiment, in which the vector with two targets was used, resulted in 0% editing efficiency, whereas the second experiment, in which only one target was used, had 7.3% editing efficiency. It is worth mentioning that two T0 edited plants, which survived and gave seeds, both had one edited allele and one wildtype allele. 

Since three different targets were used for the editing of *MtNF-YB10*, it is unlikely that the low frequency of editing is due to the low effectiveness of these targets. Therefore, we can hypothesize that loss of the *MtNF-YB10* function may be detrimental for plants. We did not perform a thorough phenotypic analysis of plants with homozygous loss of the *MtNF-YB10* function, but, according to our results, the germination rate was lower in *mtnf-yb10* plants, and the seedling root length was reduced. Apart from these features, simple visual observation did not detect any serious morphological or developmental abnormalities in mutant plants in vivo. 

However, the capacity of *mtnf-yb10* plants for SE in vitro was drastically reduced (Figure 6). Considering that, it is possible to infer that mutations in the *MtNF-YB10* gene specifically hamper in vitro regeneration from callus, and most plants regenerated after transformation with a CRISPR plasmid managed to repress Cas9 or guide RNA synthesis. Therefore, in the T0 generation, we mostly observed either unedited plants or, in rare cases, plants with a single edited allele.

For *LEC1* and its orthologs in different plant species, specific effects of ectopic expression were described. For example, *LEC1* overexpression in *A. thaliana* induces SE, inhibits germination [33], stimulates seed storage protein accumulation, fatty acid biosynthesis [41], etc. Similarly, *ZmLEC1* overexpression increases seed oil content and inhibits seed germination in *Z. mays* [30]. In *Oryza sativa*, ectopic expression of *OsNF-YB7 (OsLEC1)* leads to abnormal development of leaves and inflorescences, as well as to the “pseudovivipary” phenomenon when some spikelets are transformed into the plantlets [31], whereas overexpression of its close paralog *OsNF-YB9* inhibits vegetative growth and seed development [42]. For *MtNF-YB10*, the overexpression effects were evaluated in T0 calli so far, and no effect on the in vitro regeneration was found [38].

Loss of function of *LEC1* results in many abnormalities in plant development, including the loss of seed desiccation tolerance, leaf-like cotyledons, etc. [33]. To the best of our knowledge, the loss-of-function mutants for LEC1-type genes have been described only for two species, *A. thaliana* and *O. sativa*. In both cases, two LEC1-type paralogs were identified, *LEC1* and *L1L* for *A. thaliana* and *OsNF-YB7* and *OsNF-YB9* for *O. sativa*. Moreover, in both species, knockout or knockdown of these paralogous genes leads to different effects. While *lec1 A. thaliana* mutants, among other issues, demonstrate seed desiccation intolerance [33], silencing of the *L1L* gene results in embryo development arrest even before seed desiccation [43]. In rice, loss of *OsNF-YB9* function is not lethal and primarily affects seed shape [42]. At the same time, loss of *OsNF-YB7* function leads to desiccation intolerance, which is lethal for seeds unless they are harvested and germinated before desiccation [37]. These differences in mutation effects are likely related to different expression patterns. For example, *L1L* expression under the control of the *LEC1* promoter can complement the *lec1* mutation in *A. thaliana* [43]. Moreover, *Daucus carota*, *Z. mays*, and *O. sativa* LEC1-type genes were able to complement the loss-of-function *lec1* mutation in *A. thaliana* [30,42,44], demonstrating the conservation of these genes even between distant species.

Since we did not observe any deleterious developmental defects in homozygous *mtnf-yb10* mutants, we can suggest that during zygotic embryogenesis, this gene acts mostly redundantly with its closest paralogue, *MtNF-YB3*, which also belongs to the LEC1-type group. Indeed, these genes both are specifically expressed in seeds and during SE in *M. truncatula* [38]. It would be intriguing to obtain plants with the *MtNF-YB3* loss-of-function mutation and compare the functions of these two *LEC1* orthologs in *M. truncatula*.

Several non-edited plants carrying *Cas9-gRNA* cassettes yielded viable seeds, and we hypothesized that editing might occur in some of the T0 plants containing the cassette for editing. A number of cases have been described where unedited transgenic plants with a *Cas9-gRNA* cassette insertion produced offspring with edited alleles. This is associated with the resumption of *Cas9-gRNA* cassette expression in the next generation. For example, new editing events were discovered in the transgenic generation T1 (provided there was no editing in T0) in wheat for one of the analyzed genes [45] and in sorghum [46] and in the T2 generation in rapeseed [47]. We also analyzed the genotype of T1 plants obtained from non-edited T0 plants, which contained transgenic cassettes for *MtNF-YB10* editing, but no editing events were found. In recent research, treatment with nicotinamide, a histone deacetylase inhibitor, was shown to increase the frequency of *GUS* transgene editing in T2 wheat plants obtained from transgenic unedited T1 plants [48]. It would be intriguing to examine whether such a treatment could also be implemented for the editing of *MtNF-YB10* in *M. truncatula.*

Together, the results obtained suggest divergent functions of *LEC1* orthologs and make it possible to investigate potential specific *MtNF-YB10* functions.

## 4. Materials and Methods

### 4.1. Plant Material and Bacterial Strains

Plants of the *M. truncatula* R108 line, derived from ecotype 108-1 [49], were used in this study. Seeds of the R108 line were provided by colleagues from the Samuel Roberts Institute (Ardmore, OK, USA). The AGL1 strain of *Agrobacterium tumefaciens* (*Rhizobium radiobacter*) was used to transform *M. truncatula* plants. The *Escherichia coli* TOP10 strain was used to obtain genetic constructs. 

### 4.2. Plant Cultivation Conditions

Sterilization and germination of *M. truncatula* seeds were performed as described in [38]. Plants were grown in soil and in in vitro conditions at 21–24 °C, 16 (light)/8 (dark) photoperiod. For growth in the growth chambers, Terra Vita (Nord Pulp, Saint-Petersburg, Russia) soil mixed with vermiculite (3:1) was used. 

Plant transformation was performed as described in [50,51]. Briefly, young leaves were taken from sterile plants approximately 30 days after germination. From each complex leaf, separate leaflets were taken and wounded with several scalpel cuts at the abaxial side. Explants were incubated for 15 min in liquid Agrobacterium infiltration medium (1/2 modified PCI-4 medium [49] with 18 µM 2,4-dichlorophenoxyacetic acid (2,4-D), 2.22 µM 6-benzylaminopurine (BAP), and 200 µM acetosyringone) containing resuspended agrobacteria with the desired construct. The OD600 of agrobacterial suspension in the infiltration medium was 0.3. After infiltration, explants were placed abaxial side up on the solid co-cultivation medium—modified PCI-4 medium [49] with 18 µM 2,4-dichlorophenoxyacetic acid (2,4-D), 2.22 µM 6-benzylaminopurine (BAP), and 200 µM acetosyringone. After 2 days, explants were transferred to the selective medium for callus formation (CIM)—modified PCI-4 medium [49] with 18 µM 2,4-dichlorophenoxyacetic acid (2,4-D), 2.22 µM 6-benzylaminopurine (BAP), 25 mg/L hygromycin, 250 mg/L cefotaxime, and phytagel ((5 g/L) instead of agar) in darkness. Explants were cultivated on such medium for 30–65 days; during this period, they were transferred on the fresh medium approximately every 14 days. Then, developed calli were cultivated for about 25–30 days on the SE induction medium (SEIM), which was identical to the callus induction medium but did not contain 2.4-D and included agar (10 g/L) instead of phytagel. At this stage, calli were cultivated in the 16 (light)/8 (dark) photoperiod. After that, transfers to the SE induction medium were continued, but BAP concentration was gradually reduced with every transfer on the fresh medium (from 1.11 µM to 0.555 µM and, finally, 0 µM). When 1–2 true leaves were formed, the regenerants were transferred to the germination medium—modified PCI-4 medium [49] without hormones and antibiotics—and then, after 10–14 days, to the rooting medium—half-strength modified PCI-4 medium [39] without hormones and antibiotics. When the explants formed roots, they were transferred to modified Fahraeus medium [52,53] for 10–14 days and then to the soil for additional growth to obtain seeds.

Evaluation of the capacity for SE was performed as follows: Plants of the R108 and *mtnf-yb10* lines were grown in vitro conditions on modified Fahraeus medium [52,53] for 24 days. After that, parts of plant petioles closest to the leaf blade, about 5 mm in length, were excised. Several scalpel cuts were made on such explants, and after that, they were put on the medium similar to the CIM medium described above but without cefotaxime and hygromycin. Explants were cultivated on this medium in the dark, and every 8–17 days they were transferred to the same fresh medium. After 39 days, when most of the explants formed calli, they were transferred to light conditions on the medium similar to the SEIM medium but without any antibiotics and hormones. They were cultivated for 29 days on this medium; during this period, one transfer onto the same fresh medium at the 13th day of cultivation in light conditions was performed. After that, the calli weight was measured, and the somatic embryo number was counted for each explant, as described in [53]. 

Evaluation of seedling root length in wt and *mtnf-yb10* plants was performed for seedlings grown on modified Fahraeus medium [52,53], at 10 day after germination. Statistical analysis of obtained results was performed using the R environment [54] and packages “ggpubr” [55], “dplyr” [56], “gsubfn” [57], “ggplot2” [55], “ggtext” [58], “glue” [59], “vcd” [60], and “stringr” [61].

### 4.3. Microorganisms Cultivation Conditions

*E.coli* and *A. tumefaciens* bacteria were cultivated and transformed as described in [38].

### 4.4. Construction of Vectors for CRISPR/Cas9 Editing

gRNA targets of the investigated *MtLEC1* gene were selected using the CRISPR-P 2.0 program [40]. Lists of off-targets (DNA sequences in the investigated genome that could potentially be edited by the selected target) were obtained using the free online service Cas-OFFinder [62]. In order to select the most dangerous off-targets, an R script was used [63]. 

For CRISPR/Cas editing, we used pHSE401, a gift from Qi-Jun Chen (Addgene plasmid #62201; https://www.addgene.org/62201/ (accessed on 1 September 2023); RRID:Addgene_62201). pHSE401 is a binary vector with a hygromycin resistance gene as a selectable marker derived from pCAMBIA1300. For editing vector obtaining, pCBC-DT1T2 was also used as a PCR matrix. This plasmid was a gift from Qi-Jun Chen (Addgene plasmid #50590; https://www.addgene.org/50590/ (accessed on 1 September 2023); RRID:Addgene_50590).

Cloning of gRNA genes into the plasmid pHSE401 was performed using the Golden Gate method through Eco31I sites according to [39]. Phusion DNA polymerase (Thermo Fisher Scientific, Waltham, MA, USA) was used for PCR for gRNA gene amplification. The FastDigest Eco31I restriction enzyme and T4 DNA ligase (Thermo Fisher Scientific, Waltham, MA, USA) were used for cloning according to the manufacturer’s instructions.

### 4.5. Plant Genotyping

For genotyping, DNA of regenerants and their progeny was isolated according to the modified Edwards et al. method [64]. Briefly, young leaves were collected in tubes and ground with a pestle. A total of 400 µL of extraction buffer (200 mM Tris-HCl, 250 mM NaCl, 25 mM EDTA, 0.5% *w*/*v* sodium dodecyl sulfate) was added, and the samples were centrifuged for 5 min at 12,000 rpm. A total of 300 µL of the supernatant was taken into a new tube. Then, 300 µL of isopropanol was added, and the samples were mixed by inverting 5 times. The samples were incubated at −20 °C for 30 min and centrifuged for 5 min at 12,000 rpm. The supernatant was removed, and the precipitate was washed with 500 µL of 75% ethanol. The washed precipitate was dried under air flow in a laminar flow hood for 5–10 min and dissolved in 100 µL of water.

Alternatively, DNA for genotyping was isolated with the DNeasy kit (Qiagen, Hilden, Germany), according to the manufacturer’s instructions.

Isolated DNA was amplified by PCR with Polymerases Phusion (Thermo Fisher Scientific, Waltham, MA, USA), Q5 MasterMix (New England Biolabs, Ipswich, MA, USA), or iProofHigh Fidelity DNA Polymerase (Bio-Rad Laboratories, Hercules, CA, USA), according to the manufacturer’s instructions. Primer sequences are presented in Table A1.

Sanger Sequencing of the amplified fragments was performed in the Resource Centers for Gene Engineering at the Sirius University of Science and Technology and in the Research Resource Center for Molecular and Cell Technologies at Saint-Petersburg State University. In order to detect edited allele sequences, allele separation was performed with the online tool ICE Analysis, 2019, v3.0, Synthego [date accessed—15 September 2023]. In some cases, allele sequences were obtained by cloning PCR fragments into the pAL2-T linearized vector (Evrogen, Moscow, Russia), followed by sequencing plasmids from separate clones using the standard primer M13for.

## 5. Conclusions

In this study, we obtained *Medicago truncatula* plants with the loss of the *MtNF-YB10* function using CRISPR/Cas9 technology. The efficiency of the *MtNF-YB10* editing was quite low, which may be related to the role of this gene in regeneration processes. The germination rate and seedling root length were reduced in *mtnf-yb10* mutants, but we did not observe any deleterious morphological abnormalities in such plants. Interestingly, the capacity for somatic embryogenesis and callus formation in vitro was greatly reduced in *mtnf-yb10* mutants. Together, the results obtained suggest the divergent functions of *LEC1* orthologs and make it possible to investigate potential specific *MtNF-YB10* functions.

## Figures and Tables

**Table 1:** Results of allele sequence analysis of T0 edited plants.

T0 Plant	Allele 1	Allele 2	Seeds
*mtnf-yb10-1*	loss of 11 nucleotides in the target site	missense mutation in the 39th nucleotide (A39G) leading to the amino acid change I13M	No
*mtnf-yb10-* *25*	loss of 10 nucleotides	wt	Yes
*mtnf-yb10-* *31*	insertion of one nucleotide	wt	Yes

**Table 2:** Results of allele sequence analysis of T1 edited plants.

T1 Plant	Allele 1	Allele 2	Seeds
*mtnf-yb10-* *25-1*	loss of 10 nucleotides	wt	No
*mtnf-yb10-* *25-2*	loss of 10 nucleotides	loss of 10 nucleotides	Yes
*mtnf-yb10-* *31* *-1*	insertion of one nucleotide	wt	Yes

## Data Availability

Data is contained within the article.

## Appendix A

**Table A1:** Primers used in the experiments.

Primer Name	Sequence	Experiment Type
CRISPR Oligo MtNF-YB10 target 3 for	ATTGGTTTGCTATTGGCATGTAC	Cloning for editing
CRISPR Oligo MtNF-YB10 target 3 rev	AAACGTACATGCCAATAGCAAAC	Cloning for editing
MtNF-YB10_DT1-BsF	ATATATGGTCTCGATTGATACGAATCACGTTTGCTATGTT	Cloning for editing
MtNF-YB10_DT1-F0	TGATACGAATCACGTTTGCTATGTTTTAGAGCTAGAAATAGC	Cloning for editing
MtNF-YB10_DT2-R0	AACATTGCCCATAGTATATCTTCAATCTCTTAGTCGACTCTAC	Cloning for editing
MtNF-YB10_DT2-BsR	ATTATTGGTCTCGAAACATTGCCCATAGTATATCTTCAA	Cloning for editing
M13 for	GTAAAACGACGGCCAGT	Sequencing
M13 rev	CAGGAAACAGCTATGAC	Sequencing
MtNF-YB10_Medtr1g039040_target1_for	GGTTTTTCCAAATTATATATTGAGATTTT	Genotyping and sequencing
MtNF-YB10_Medtr1g039040_target1_rev	CTTATGTATTCGGATACACATTCT	Genotyping and sequencing
MtNF-YB10_Medtr1g039040_targets_rev	GGAAAAGAAGGAGGCAAAGCA	Genotyping and sequencing
SpCas9 for	CCTGGAGGCGAAGGGCTACAA	Transgene identification
SpCas9 rev	GAAGTTCACATACTTGGACGGCAGA	Transgene identification
MtH3L_Medtr4g097170_for	CTTTGCTTGGTGCTGTTTAGATGG	PCR positive control
MtH3L_Medtr4g097170_rev	ATTCCAAAGGCGGCTGCATAPCR positive control	PCR positive control
